# Supplementary material for: A comprehensive resource for Bordetella genomic epidemiology and biodiversity studies
Source: Nat Commun. 2022 Jul 1;13:3807. doi: 10.1038/s41467-022-31517-8 (PMC9249784; doi:10.1038/s41467-022-31517-8)
Supplement: Supplementary file 1 — Supplementary Information File [file 41467_2022_31517_MOESM1_ESM.pdf]

Supplementary Information

A comprehensive resource for *Bordetella* genomic epidemiology and biodiversity studies

Author list

Sébastien Bridel[1,#] & Valérie Bouchez[1,2,#], Bryan Brancotte[3], Sofia Hauck[4], Nathalie Armatys[1,2], Annie Landier [1,2], Estelle Mühle[5], Sophie Guillot[1,2], Julie Toubiana[1,2,6], Martin C.J. Maiden[4], Keith A. Jolley[4], Sylvain Brisse[1,2,*]

Affiliations

1 Institut Pasteur, Université Paris Cité, Biodiversity and Epidemiology of Bacterial Pathogens, Paris, France.

2 National Reference Center for Whooping Cough and other Bordetella Infections, Institut Pasteur, Paris, France.

3 Institut Pasteur, Université Paris Cité, Bioinformatics and Biostatistics Hub, F-75015 Paris, France

4 Department of Zoology, University of Oxford, 11a Mansfield Road, Oxford, OX1 3SZ, United Kingdom

5 Collection de Institut Pasteur, Institut Pasteur, Université Paris Cité, Paris, France

6 Université Paris Cité, Department of General Pediatrics and Pediatric Infectious Diseases, Hôpital Necker–Enfants Malades, APHP, Paris, France

# Equal contribution

* **Correspondence**: **Sylvain Brisse**: Institut Pasteur, Biodiversity and Epidemiology of Bacterial Pathogens, 28 rue du Docteur Roux, F-75724 Paris, France. Phone: +33 1 45 68 83 34; E-mail: sylvain.brisse@pasteur.fr; ORCID Number: 0000-0002-2516-2108

Contents :

**Supplementary figures**	page 2
**Supplementary data**	page 10
**Supplementary notes**	page 11

Supplementary references page 15

Supplementary Figures

Supplementary Fig. 1: ANI heatmap of the *Bordetella* genus

Heatmap showing ANI values among *Bordetella* species and yet-unnamed genomic species (G. S.). ANI values are colored from 75 (red) to 100 (blue). The heatmap was computed based on the ANI values matrix and ordered by internal classical hierarchical clustering of those values. ANI threshold for genomic species definition is 95%. Source data are provided as a Source Data file.

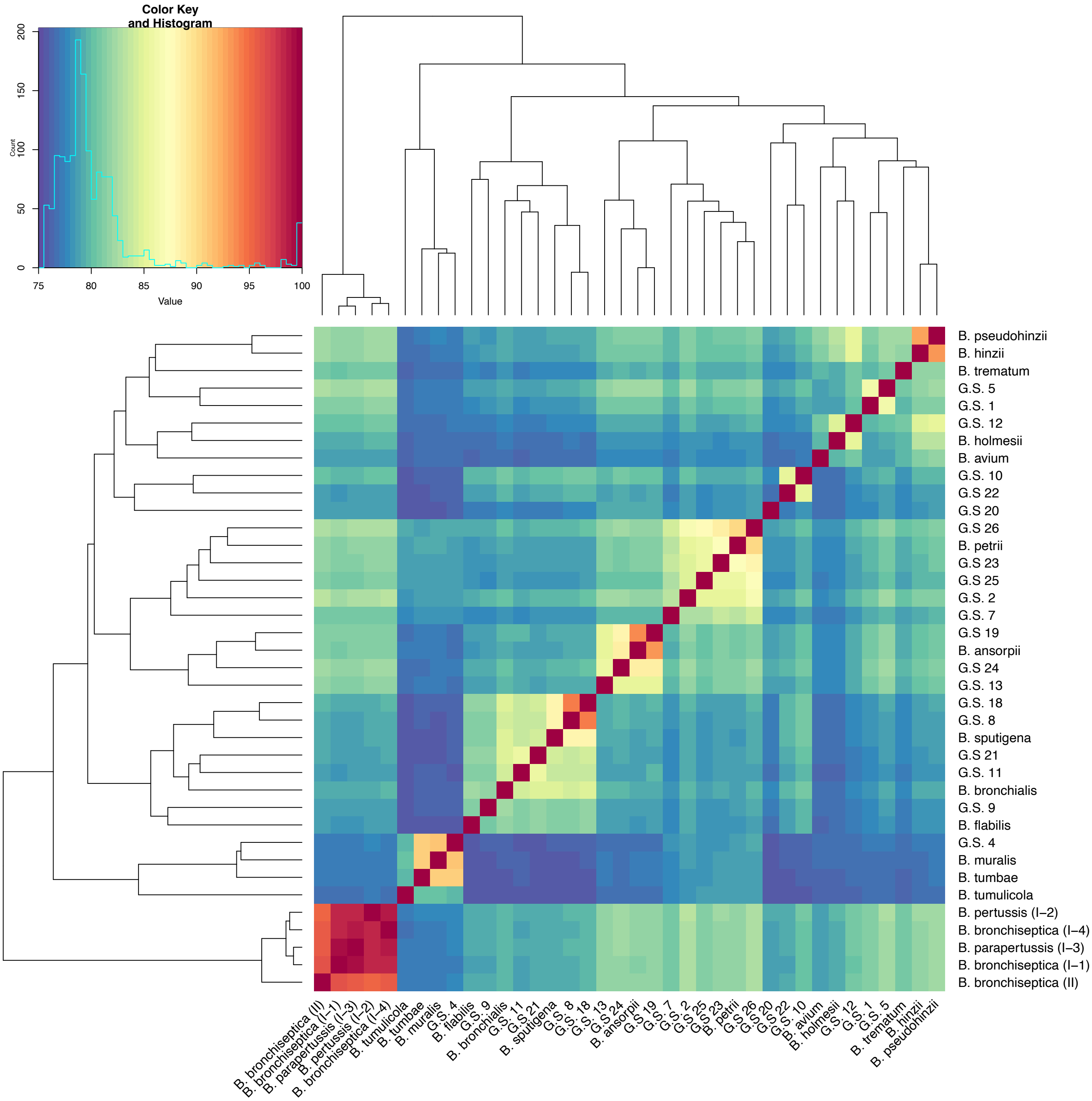

Supplementary Fig. 2: Genome-based phylogeny of the *Bordetella* genus

As for Figure 1, the analysis was performed with 92 *Bordetella* genomes. The phylogenetic tree was built using JolyTree v2.1 and is rooted using *Ralstonia* species. Bootstrap values are indicated using light blue circles (see key). Genomic species are labelled G.S. An interactive iTOL version of the tree can be accessed at: https://itol.embl.de/shared/1l7Fw0AvKOoCF.

Source data are provided as a Source Data file.

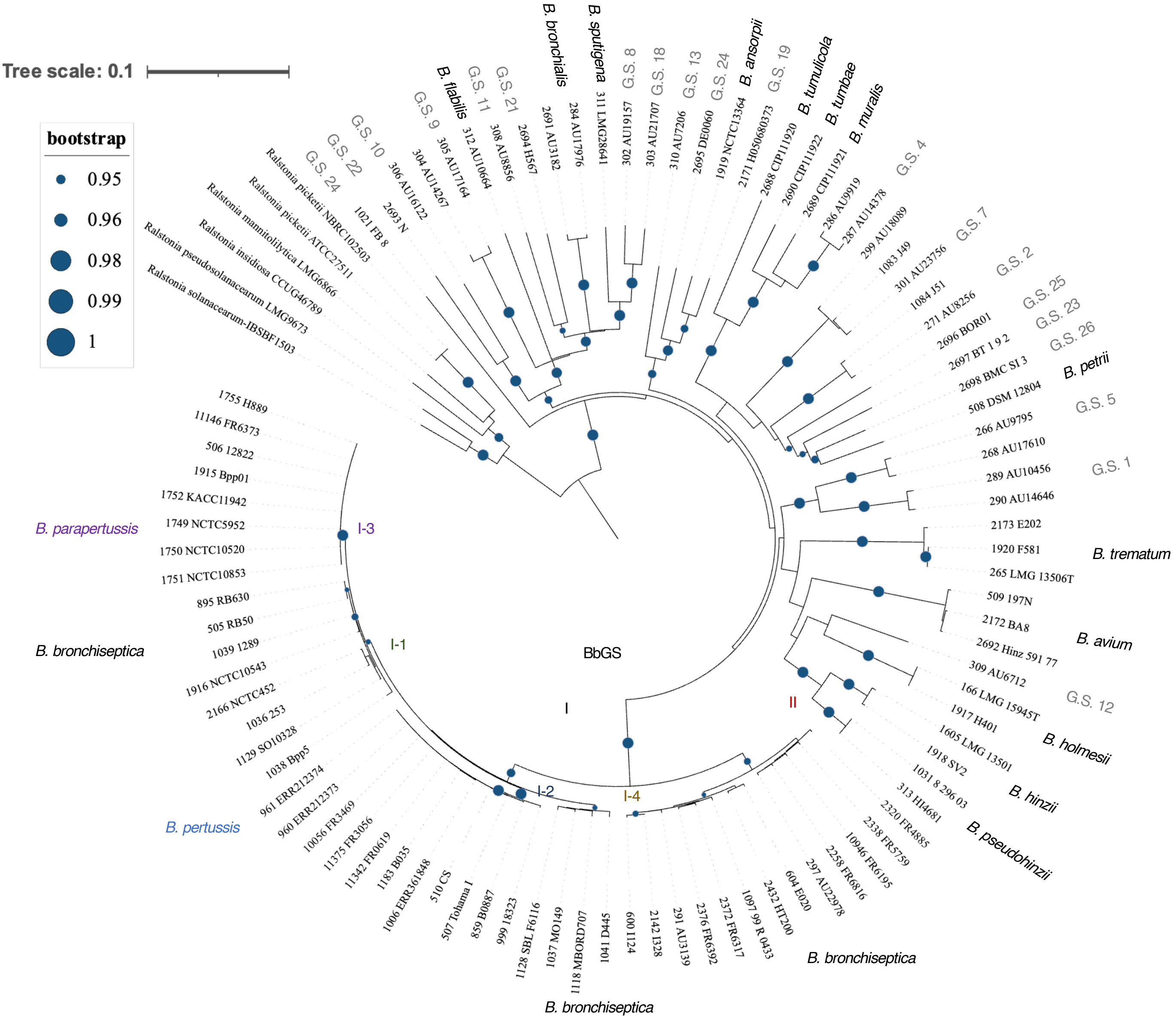

Supplementary Fig. 3: Phylogeny of the *B. bronchiseptica genomic species*

The analysis was performed with the same 190 *B. bronchiseptica* genomes and representatives of the phylogenetic diversity of *B. pertussis* and *B. parapertussis,* as in Figure 2. The recombination-purged concatenated multiple sequence alignment of 1,415 core gene loci (*cgMLST_genus* scheme) was used. The tree is rooted on lineage II, which is the most divergent clade. Isolates names are colored according to their lineage or sublineages (I-1, green; I-2 [B. pertussis], blue; I-3 [B. parapertussis], red; I-4 brown; II, purple). Boostrap values equal to 100 are indicated on the branches by a blue circle. An interactive iTOL version of the tree can be accessed at: https://itol.embl.de/shared/1l7Fw0AvKOoCF.

Source data are provided as a Source Data file.

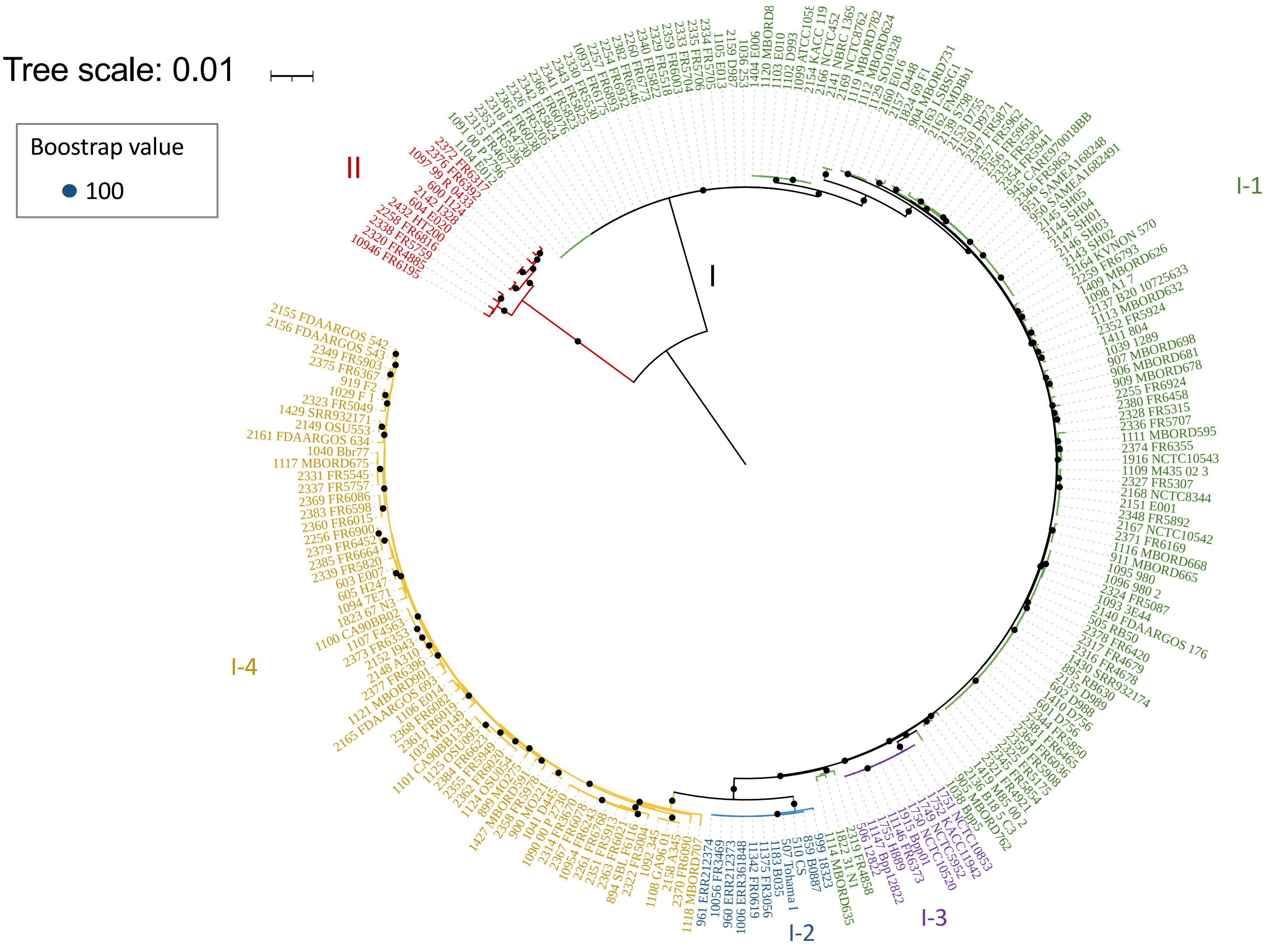

Supplementary Fig. 4: Comparison of the allele call rates of two cgMLST schemes on classical *Bordetella* isolates and on *B. bronchiseptica* lineage II.

The boxplots represent the distribution of the fraction of uncalled loci for the two cgMLST schemes: *cgMLST-pertussis* and *cgMLST-genus*. Boxplots are colored according to lineage or sublineages (see key). The lower and upper hinges of the box plots correspond to the first and third quartiles (the 25th and 75th percentiles); horizontal bar represent the median; data beyond the end of the whiskers are called "outlying" points and are plotted individually (please see *geom_boxplot* function of ggplot2 for details). 211 biologically independent genomes were analyzed

(Online documentation: https://ggplot2.tidyverse.org/reference/geom_boxplot.html)

Source data are provided as a Source Data file.

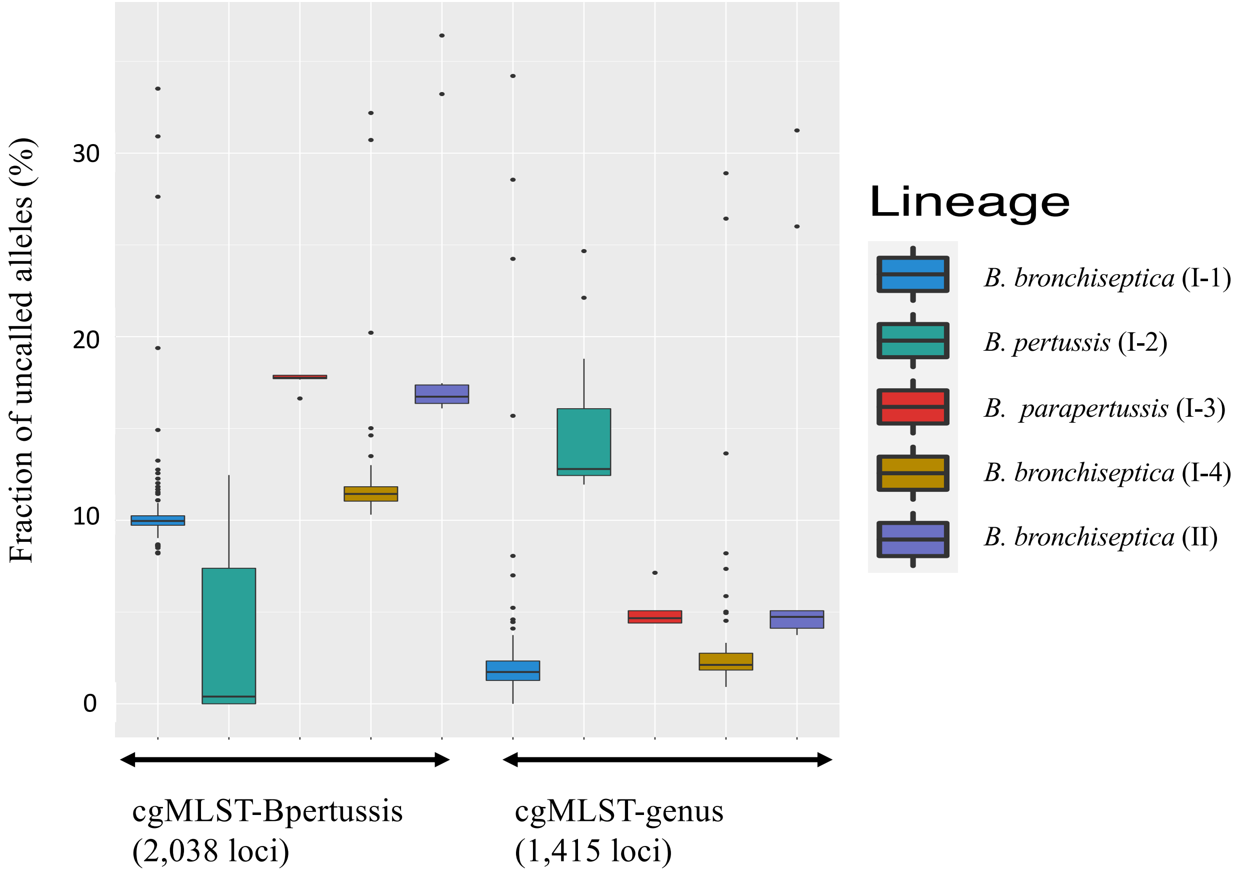

Supplementary Fig. 5: Virulence allele profiles within the *Bordetella bronchiseptica* genomic species. Same tree as in main Figure 2, with branch lengths. The outer circles represent allele variation at loci of the different virulence schemes, labeled by their BIGSdb scheme id: *Bp* Vaccine antigens (id 8), Phase biology genes (id 9), Other Toxins (10), T3SS (id 11), Autotransporters (id 12). Each color is specific to an allele number; across loci, similar color panels were used for each lineage or sublineage to illustrate lineage specificity of allele variation.

An interactive iTOL version of the tree can be accessed at: https://itol.embl.de/shared/1l7Fw0AvKOoCF.
Source data are provided as a Source Data file.

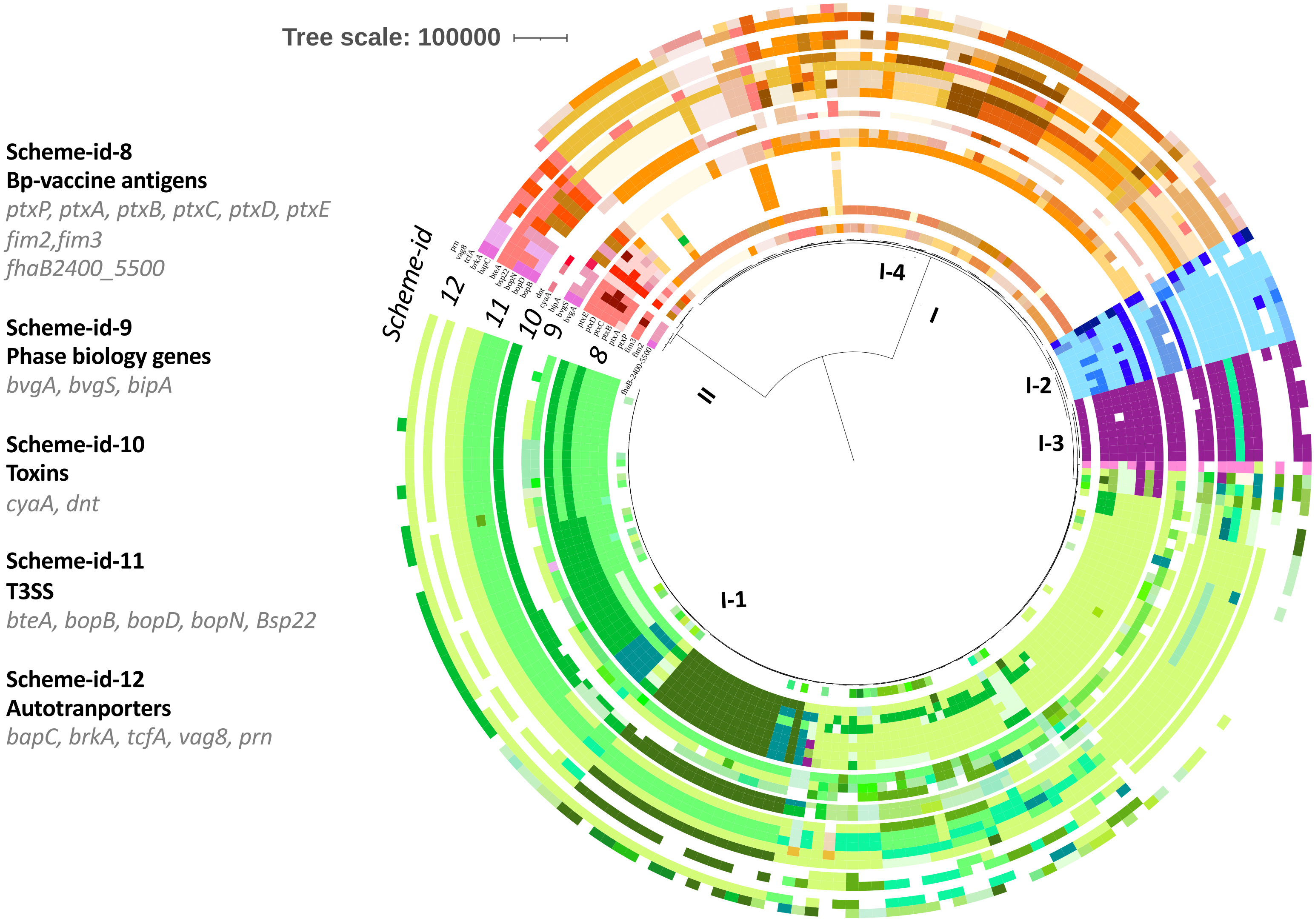

Supplementary Fig. 6: Geographic repartition of *ptxP* and *fhaB* alleles of *B. pertussis* isolates.

Isolates correspond to BIGSdb project id 25 and are colored according to country of origin (see key). We compared the geographical origin of isolates with *ptxP1* (panel A-1) or *ptxP3* (panel A-2) alleles. For temporal homogeneity, the dataset comprises only the isolates of the *ptxA1* clade, which were all collected after 2008. **B-1 and B-2**: Geographical origin of *fhaB1* and *fhaB3* isolates; most of the latter are resistant to macrolides.

Source data are provided as a Source Data file.

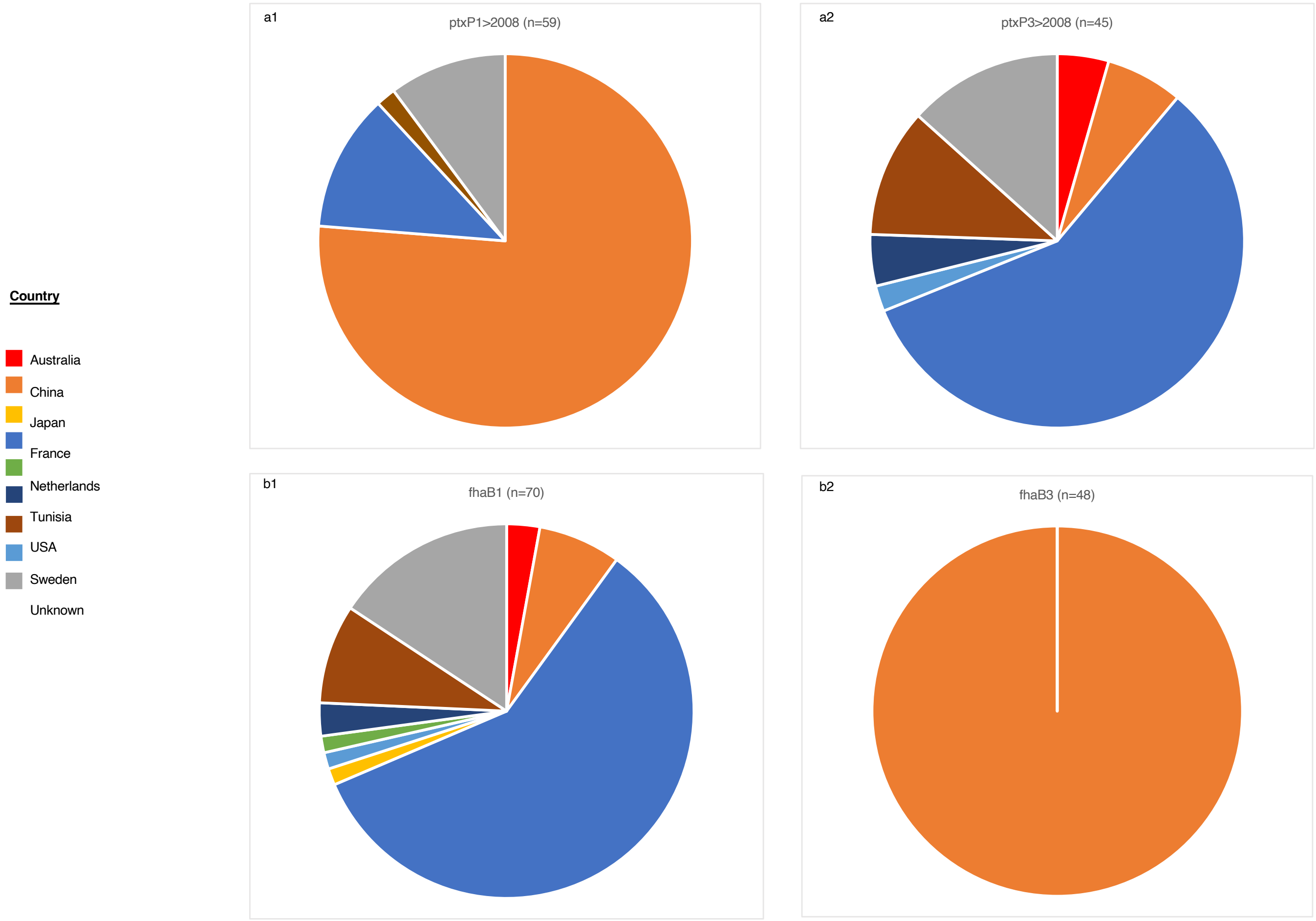

Supplementary Fig. 7: GrapeTree visualization of 1949 BbGS public genomes.

The minimum spanning tree was constructed using GrapeTree from within the BIGSdb platform, based on cgMLST_genus scheme loci. Branches longer than 1276 cgMLST mismatches are hidden. The network allows the visualization of 5 groups within the BbGS: *B. bronchiseptica* I-1 (green), *B. pertussis* (I-2, blue), *B. parapertussis* (I-3, purple), *B. bronchiseptica* I-4 (yellow) [which were previously described using MLST by Diavatopoulos *et al.* 2005 as MLST complexes I, II, III and IV, respectively][1], and *B. bronchiseptica* lineage II (red). *B. pertussis* lineage I described in Bart *et al.*[2] is highlighted on the figure by a dotted circle, and the unique ovine *B. parapertussis* genome is colored in pink.

Source data are provided as a Source Data file.

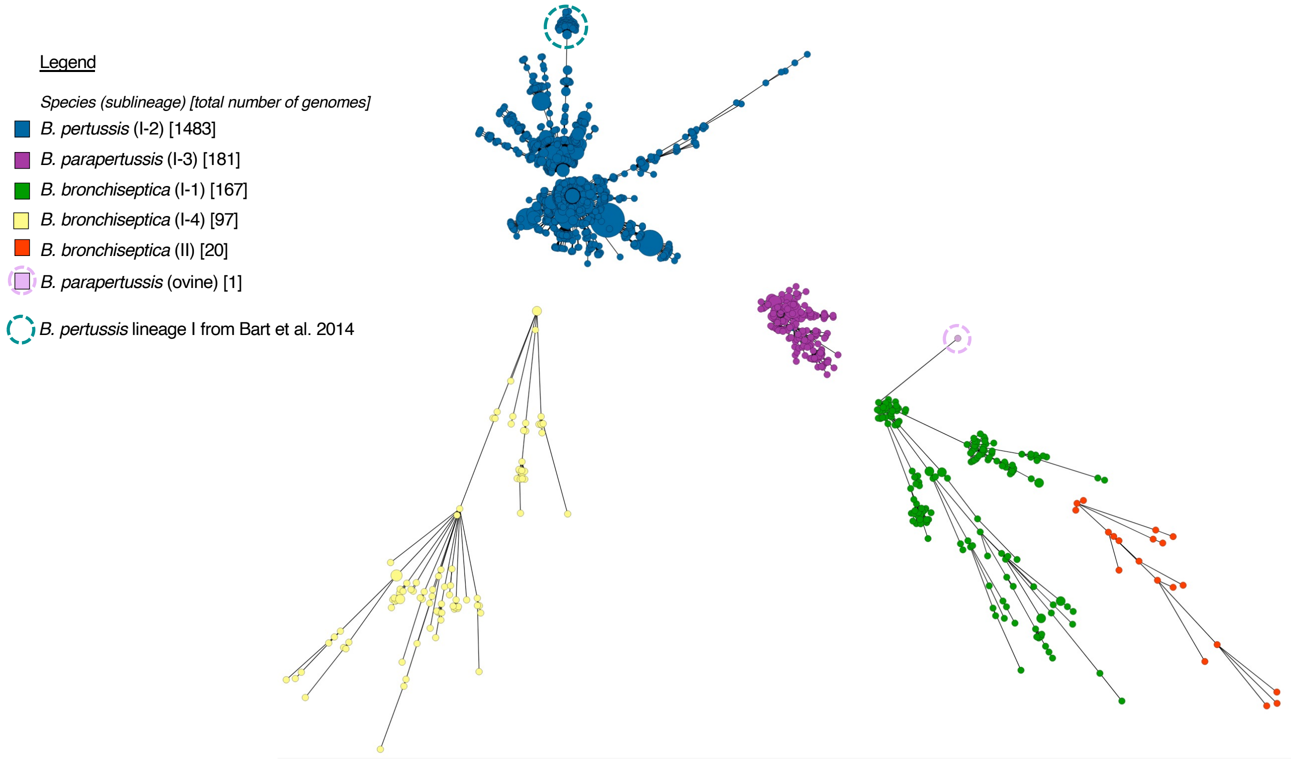

Supplementary Fig. 8: *nrdA* phylogenetic tree of BIGSdb public genomes

The tree was made using the neighbor-joining method within BIGSdb and was midpoint rooted and annotated using iTOL, called directly from the BIGSdb platform. Public genomes were used, but only some representatives of the BbGS were selected (same as those in Figure 1: project ID23; Supplementary Data 4). Isolate LUAb4 (ID 2773) was recently described as a putative novel *Bordetella* species "*B. atropi*" [3] and was included only in this analysis (other analyses were made before the release of this genome). The complete list (180 isolates) is provided in BIGSdb project "Public-nrdA project" (BIGSdb project ID 29). "*B. atropi*" and the genomic species appear in grey. *B. bronchiseptica* lineages are colored (*B. bronchiseptica* I-1: green, *B pertussis*: blue, *B. parapertussis*: purple, *B. bronchiseptica* I-4: brown, *B. bronchiseptica* II: red). Isolate Bpp5 (ovine *B. parapertussis*) is highlighted in light purple. An interactive iTOL version of the tree can be accessed at: https://itol.embl.de/shared/1l7Fw0AvKOoCF.

Source data are provided as a Source Data file.

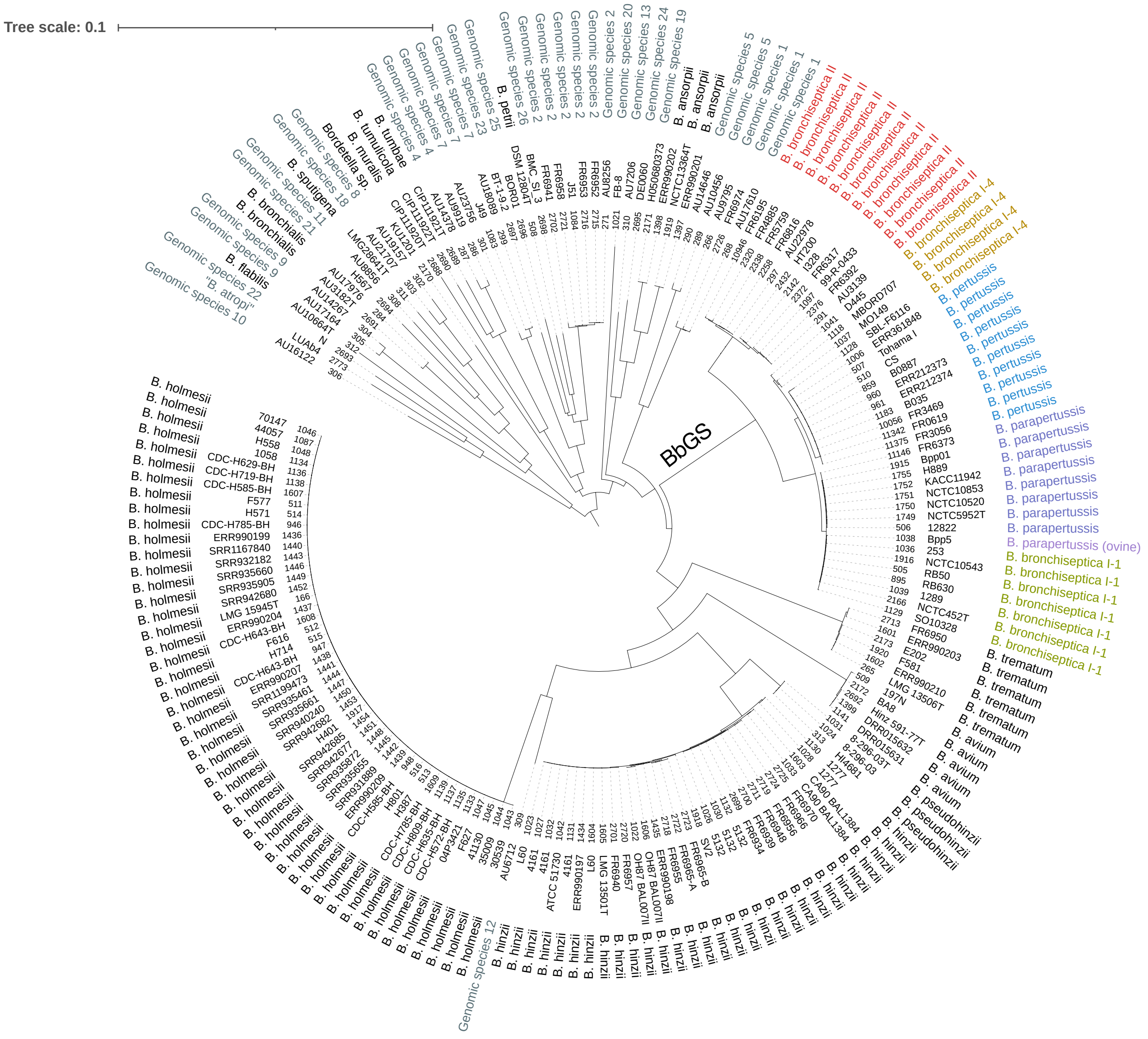

Tree scale: 0.1
BbGS

Supplementary Data

Supplementary Data 1: ANI values across the *Bordetella* genus
Supplementary Data 2: Full list of isolates of novel genomic species
Supplementary Data 3: cgMLST genome coverage
Supplementary Data 4: *Bordetella* genomes list and accession numbers
Supplementary Data 5: Correspondence between former alleles from Oxford's PubMLST BIGSdb database, and the current alleles in the merged database
Supplementary Data 6: Main alleles of the virulence-related schemes observed in the BbGS lineages

Supplementary notes: Variation of vaccine antigens and virulence-associated genes

The main alleles of the loci of these schemes are summarized in **Supplementary Data 6**. We here describe the presence or absence of these genes, and the distribution of their alleles, within *B. bronchiseptica (Bbs)* and *B. pertussis (Bp)*.

1. Variation within *B. bronchiseptica*

Supplementary Fig. 5 provides a visual illustration of allele variation of the different genotyping schemes presented below.

Bp-vaccine antigens scheme (fim2, fim3, ptxP, ptxA, ptxB, ptxC, ptxD, ptxE, fhaB-2400_5550)

Even though pertussis toxin (PT) is known to be only produced by *Bp* isolates, the loci for PT promoter and subunits (*ptxP*, *ptxA*, *ptxB*, *ptxC*, *ptxD*, and *ptxE)* were present across most of the BbGS members. When present, alleles of these loci were specific for each lineage, *i.e.,* uniquely observed within a single lineage.

A notable exception was observed for 58 of 63 isolates from sublineage I-4, in which no alleles of the 6 PT-related loci were detected. This is in agreement with previous results underlying *ptxABCDE* absence in Bbr77 and MO149 isolates from sublineage I-4 [4]. Furthermore, we noticed that isolates of *Bbs* lineage II did not have alleles for the *ptxP* locus. This was true with the initial scan parameters (i.e. 90% identity, 90% alignment with the type allele) as well as when releasing these scan parameters to 50%. Regarding fimbriae, alleles were only detected in lineage I-2 (*Bp*) for locus *fim2* using our standard parameters. However, when releasing the scan parameters, we obtained partial fim2 matches for 78 isolates of lineages I-1, I-3, I-4 and 2, with identity percentage ranging from 78% to 97%.

For locus *fhaB* (2400-5550 region), no alleles were captured for 71 isolates, 67 of them belonging to sublineage I-1. Alleles were missing in 16/67 genomes but a partial match (from 1591 bp to 3099 bp over 3151 bp) was found when releasing scan parameters for the remaining isolates (51/67), with a high identity score (from 98 to 100%).

Other toxins scheme

For the two loci *cyaA* and *dnt*, included in the “Other toxins” scheme, we observed either sequence polymorphism or variation in the presence of these genes.

Gene *cyaA* encodes adenylate cyclase, an important toxin produced by isolates of the BbGS [5,6]. No alleles could be assigned for 32 isolates of lineages I-1 and 6 isolates of lineage II, consistent with previous findings: for strain 253 (BIGSdb ID: 1036) of lineage I-1 [6,7], the *cyaA* gene is replaced by a *ptp* operon [6]. Interestingly, in 5/6 isolates from lineage II (as for strains HT200 [8], BIGSdb ID: 2432), no alleles were found even when releasing the scan parameters.

The dermonecrotic toxin (DNT) is involved in turbinate atrophy and bronchopneumonia in pigs [9]. All isolates collected from pigs in our dataset were found in sublineage I-1 and had alleles at the *dnt* locus (as for example in strain S798 used to investigate DNT expression in Okada *et al* [10] (BIGSdb ID: 2139). However, we observed the absence of *dnt* alleles for isolates of lineage I-4, and for the ovine *B. parapertussis*, consistent with previous reports for lineage I-4 strains Bbr77 and D445 (BIGSdb ID 1040 and 1041) [7]. In the same way, strain HT200 of *Bbs* lineage II had no allele for *dnt* even when releasing scan parameters [8].

Autotransporters scheme

Autotransporters play an important role in *Bordetella* virulence. Among all autotransporters identified so far [11], five major autotransporters were included in the genotyping scheme: *prn*, *bapC, brkA, tcfA and vag8*.

Pertactin (Prn) is an important adhesin in the BbGS. This protein contributes to *Bbs* shedding and transmission between hosts [12]. All circulating BbGS isolates produced Prn before the use of vaccines, with a high variability among lineages due to different numbers of short repeats [13,14]. Since the introduction of acellular vaccines, an increasing number of isolates of lineages I-2 (*Bp*) and I-3 (*Bpp*) are deficient for Prn production [12,15]. This is reflected in our scanning results (Supplementary Fig. 5).

BapC (Bordetella Autotransporter Protein C) and BrkA (Bordetella Resistance to killing protein A) play a role in protecting *B. pertussis* from serum killing by complement [16,17]. Alleles were missing for *bapC* in many isolates of lineages I-1 and in all lineage I-3 (*Bpp*) isolates. In the same way, *brkA* alleles were missing in few isolates from lineage I-1 and in 8 of 10 isolates of lineage I-3 (*Bpp*).

Gene *tcfA*, coding for the tracheal colonization factor A, was only tagged for 6 of 11 isolates of sublineage I-2 (*Bp)*, consistent with literature [4,18,19].

Vag8 is an additional autotransporter involved in complement evasion [20]. Alleles were captured for isolates of lineages I-4 and I-2 (*Bp*) but were missing in some isolates of lineage I-1 and in all isolates of lineage II.

T3SS scheme

The allele variation at loci of this scheme is defined in the main text.

Phase biology genes scheme

Phase variation due to mutations in either *bvgA* or *bvgS* is frequent in *Bbs*. We found that *bvgA* and *bvgS* alleles were highly specific for each lineage or sublineage.

bipA sequence variation was described within the BbGS [21]. Even though *bipA* genes of *Bp* Tohama I and *Bbs* RB50 differ in the number of 90-amino-acid repeats and in their C-terminal sequence [21], *bipA* allele 3 was observed for isolates from Bp (sublineage I-2, comprising Tohama) and other alleles for some of sublineage I-1 (comprising RB50).

2. Variation within *B. pertussis*

As expected, the diversity of alleles of the different schemes was much lower for *B. pertussis* than within the BbGS.

Bp-vaccine antigen scheme (fim2, fim3, ptxP, ptxA, ptxB, ptxC, ptxD, ptxE, fhaB-2400_5550)

The allele variation at loci of this scheme is defined in the main text.

Other toxins scheme

Bp isolates were characterized by *cyaA* allele 4*,* except for one isolate (ID 1240_B096) with allele 43. Eight isolates had no alleles but presented a partial match of 3402 to 3424 bp vs 5121 as expected with 100% identity to *cyaA* gene.

Regarding the *dnt* locus, Bp isolates had allele 1, except for 3 isolates with allele 4 (IDs 532, 533 and 10408) and another with allele 34 (ID 2089). Isolate FR6006 (ID 10220) had no allele for *dnt*.

Autotransporters scheme

Two *prn* alleles were predominant (alleles 1 and 2) in our dataset; as we matched our allele nomenclature with denominations present in the literature, this is consistent with the well-known shift from prn1 to prn2 that arose in the WCV period and increased in frequency in the ACV period [22]. We also detected 3 isolates with different alleles (prn-3, 7 and 9). In addition, because of the high prevalence of PRN-deficient isolates, often due to an incomplete *prn* CDS, a large proportion of isolates had no allele for *prn*, and these were mostly *ptxP3*.

Autotransporters genes *bapC* and *vag8* were highly conserved, as all *Bp* isolates were characterized by allele 1 for *bapC* locus and allele 4 for *vag8*.

Most Bp isolates had allele 1 for *brkA*, except for one isolate with allele 64 (ID 12189, isolate B1816); 14 isolates had no called allele.

tcfA was more diverse: although most isolates had allele 2, six other alleles were found, in one isolate each (alleles 4, 5, 11, 12, 13 and 35) and nine isolates had no allele. *Bp* isolates with no *tcfA* because of the entire gene deletion have previously been reported [19].

T3SS scheme

T3SS loci were highly conserved, and as a consequence T3ST-3 was found for most isolates. The main allele for *bopB, bopD* and *bopN*, *bsp22* or *betA* loci was allele 1. Three isolates had allele 4 for *bopB* and one had allele 33 for *bteA*. In addition, 11 isolates had no allele for *bteA*. *Bp* isolates producing no *bteA* have already been evidenced [23], such as FR0145 (ID 695) due to the insertion of an IS*481* in its promoter region.

Phase biology genes scheme

Almost all *Bp* isolates had allele 1 for *bvgA* and allele 5 for *bvgS.* One isolate presented a different *bvgA* allele (IDs 10140=FR4930, allele 2). In the same way, 5 isolates had a different *bvgS* allele (alleles 4, 6, 10, 11 and 63).

bipA allele was 3 in all *Bp* isolates and other alleles were captured for some *Bbs* isolates of sublineage I-1 (comprising RB50) consistent with previous findings [24].

Supplementary references

1. Diavatopoulos, D. A. *et al.* Bordetella pertussis, the Causative Agent of Whooping Cough, Evolved from a Distinct, Human-Associated Lineage of B. bronchiseptica. *PLOS Pathogens* **1**, e45 (2005).
2. Bart, M. J. *et al.* Global Population Structure and Evolution of Bordetella pertussis and Their Relationship with Vaccination. *mBio* **5**, e01074-14 (2014).
3. Tran, T. D., Ali, M. A., Lee, D., Félix, M.-A. & Luallen, R. J. Bacterial filamentation as a mechanism for cell-to-cell spread within an animal host. *Nat Commun* **13**, 693 (2022).
4. Park, J. *et al.* Comparative genomics of the classical Bordetella subspecies: the evolution and exchange of virulence-associated diversity amongst closely related pathogens. *BMC Genomics* **13**, 545 (2012).
5. Chenal-Francisque, V., Caro, V., Boursaux-Eude, C. & Guiso, N. Genomic analysis of the adenylate cyclase-hemolysin C-terminal region of Bordetella pertussis, Bordetella parapertussis and Bordetella bronchiseptica. *Res Microbiol* **160**, 330–336 (2009).
6. Buboltz, A. M. *et al.* Replacement of adenylate cyclase toxin in a lineage of Bordetella bronchiseptica. *J Bacteriol* **190**, 5502–5511 (2008).
7. Park, J. *et al.* Comparative genomics of the classical Bordetella subspecies: the evolution and exchange of virulence-associated diversity amongst closely related pathogens. *BMC Genomics* **13**, 545 (2012).
8. Badhai, J. & Das, S. K. Genomic plasticity and antibody response of Bordetella bronchiseptica strain HT200, a natural variant from a thermal spring. *FEMS Microbiol Lett* **368**, (2021).
9. Brockmeier, S. L. *et al.* Role of the dermonecrotic toxin of Bordetella bronchiseptica in the pathogenesis of respiratory disease in swine. *Infect Immun* **70**, 481–490 (2002).
10. Okada, K. *et al.* Polymorphisms influencing expression of dermonecrotic toxin in Bordetella bronchiseptica. *PLoS One* **10**, e0116604 (2015).
11. Parkhill, J. *et al.* Comparative analysis of the genome sequences of Bordetella pertussis, Bordetella parapertussis and Bordetella bronchiseptica. *Nat Genet* **35**, 32–40 (2003).
12. Ma, L. *et al.* Pertactin contributes to shedding and transmission of Bordetella bronchiseptica. *PLoS Pathog* **17**, e1009735 (2021).
13. Boursaux-Eude, C. & Guiso, N. Polymorphism of repeated regions of pertactin in Bordetella pertussis, Bordetella parapertussis, and Bordetella bronchiseptica. *Infect Immun* **68**, 4815–4817 (2000).
14. Diavatopoulos, D. A., Hijnen, M. & Mooi, F. R. Adaptive evolution of the Bordetella autotransporter pertactin. *J Evol Biol* **19**, 1931–1938 (2006).
15. Bouchez, V., Brun, D., Dore, G., Njamkepo, E. & Guiso, N. Bordetella parapertussis isolates not expressing pertactin circulating in France. *Clin Microbiol Infect* **17**, 675–682 (2011).
16. Bokhari, H. *et al.* Molecular typing of Bordetella parapertussis isolates circulating in Pakistan. *FEMS Immunol Med Microbiol* **63**, 373–380 (2011).
17. Barnes, M. G. & Weiss, A. A. BrkA protein of Bordetella pertussis inhibits the classical pathway of complement after C1 deposition. *Infect Immun* **69**, 3067–3072 (2001).
18. Finn, T. M. & Stevens, L. A. Tracheal colonization factor: a Bordetella pertussis secreted virulence determinant. *Mol Microbiol* **16**, 625–634 (1995).
19. van Gent, M. *et al.* Characterization of Bordetella pertussis clinical isolates that do not express the tracheal colonization factor. *FEMS Immunol Med Microbiol* **51**, 149–154 (2007).
20. Marr, N., Shah, N. R., Lee, R., Kim, E. J. & Fernandez, R. C. Bordetella pertussis autotransporter Vag8 binds human C1 esterase inhibitor and confers serum resistance. *PLoS*

One **6**, e20585 (2011).
21. Fuchslocher, B., Millar, L. L. & Cotter, P. A. Comparison of bipA alleles within and across Bordetella species. *Infect Immun* **71**, 3043–3052 (2003).
22. Bart, M. J. *et al.* Global population structure and evolution of Bordetella pertussis and their relationship with vaccination. *MBio* **5**, e01074 (2014).
23. Hegerle, N. *et al.* In-vitro and in-vivo analysis of the production of the Bordetella type three secretion system effector A in Bordetella pertussis, Bordetella parapertussis and Bordetella bronchiseptica. *Microbes Infect* **15**, 399–408 (2013).
24. Fuchslocher, B., Millar, L. L. & Cotter, P. A. Comparison of bipA alleles within and across Bordetella species. *Infect Immun* **71**, 3043–3052 (2003).
