# Supplementary material for: A comprehensive resource for Bordetella genomic epidemiology and biodiversity studies
Source: Nat Commun. 2022 Jul 1;13:3807. doi: 10.1038/s41467-022-31517-8 (PMC9249784; doi:10.1038/s41467-022-31517-8)
Supplement: Supplementary file 2 — Reporting Summary [file 41467_2022_31517_MOESM2_ESM.pdf]

nature portfolio

Corresponding author(s): Sylvain Brisse

Last updated by author(s): 10/06/2022

Reporting Summary

Nature Portfolio wishes to improve the reproducibility of the work that we publish. This form provides structure for consistency and transparency in reporting. For further information on Nature Portfolio policies, see our Editorial Policies and the Editorial Policy Checklist.

Statistics

For all statistical analyses, confirm that the following items are present in the figure legend, table legend, main text, or Methods section.

n/a	Confirmed	
[ ]	[x]	The exact sample size (*n*) for each experimental group/condition, given as a discrete number and unit of measurement
[ ]	[x]	A statement on whether measurements were taken from distinct samples or whether the same sample was measured repeatedly
[x]	[ ]	The statistical test(s) used AND whether they are one- or two-sided<br>*Only common tests should be described solely by name; describe more complex techniques in the Methods section.*
[x]	[ ]	A description of all covariates tested
[x]	[ ]	A description of any assumptions or corrections, such as tests of normality and adjustment for multiple comparisons
[x]	[ ]	A full description of the statistical parameters including central tendency (e.g. means) or other basic estimates (e.g. regression coefficient) AND variation (e.g. standard deviation) or associated estimates of uncertainty (e.g. confidence intervals)
[x]	[ ]	For null hypothesis testing, the test statistic (e.g. *F*, *t*, *r*) with confidence intervals, effect sizes, degrees of freedom and *P* value noted<br>*Give P values as exact values whenever suitable.*
[x]	[ ]	For Bayesian analysis, information on the choice of priors and Markov chain Monte Carlo settings
[x]	[ ]	For hierarchical and complex designs, identification of the appropriate level for tests and full reporting of outcomes
[x]	[ ]	Estimates of effect sizes (e.g. Cohen's *d*, Pearson's *r*), indicating how they were calculated

Our web collection on statistics for biologists contains articles on many of the points above.

Software and code

Policy information about availability of computer code

Data collection	BIGSdb (v 1.35)
Data analysis	fastANI (v1.33); IQ-TREE (v2.0.6); gubbins (v 3.1.6); iTOL (v 6.5.7), BIGSdb (v 1.35), GrapeTree, JolyTree (v2.1), MAFFT (v 7.467)

For manuscripts utilizing custom algorithms or software that are central to the research but not yet described in published literature, software must be made available to editors and reviewers. We strongly encourage code deposition in a community repository (e.g. GitHub). See the Nature Portfolio guidelines for submitting code & software for further information.

Data

Policy information about availability of data

All manuscripts must include a data availability statement. This statement should provide the following information, where applicable:

- Accession codes, unique identifiers, or web links for publicly available datasets
- A description of any restrictions on data availability
- For clinical datasets or third party data, please ensure that the statement adheres to our policy

BIGSdb Pasteur : https://bigsdb.pasteur.fr
BIGSdb Pasteur, Bordetella resource: https://bigsdb.pasteur.fr/bordetella/

The sequence reads data generated in this study for Bordetella bronchiseptica isolates from France, and for B. tumulicola, B. muralis and B. tumbae type strains,

have been deposited in the European Nucleotide Archive (ENA) database, part of INSDC (NCBI/ENA/DDBJ), and is accessible under BioProject number PRJEB49946 (https://www.ebi.ac.uk/ena/browser/text-search?query=PRJEB49946).

Bordetella genomes list and accession numbers: Supplementary Data 4

Bordetella genus phylogeny dataset (92 isolates), project id 23: https://bigsdb.pasteur.fr/cgi-bin/bigsdb/bigsdb.pl?db=pubmlst_bordetella_isolates&page=query&project_list=23&submit=1
B. bronchiseptica phylogeny dataset (211 isolates), project id 24: https://bigsdb.pasteur.fr/cgi-bin/bigsdb/bigsdb.pl?db=pubmlst_bordetella_isolates&page=query&project_list=24&submit=1
B. pertussis phylogeny (124 isolates), project id 25 : https ://bigsdb.pasteur.fr/cgi-bin/bigsdb/bigsdb.pl?db=pubmlst_bordetella_isolates&page=query&project_list=25&submit=1
Bordetella public genomes dataset (2,085 isolates), project id 27: https://bigsdb.pasteur.fr/cgi-bin/bigsdb/bigsdb.pl?db=pubmlst_bordetella_isolates&page=query&project_list=27&submit=1
Bordetella genus nrdA dataset (180 isolates), project id 29: https://bigsdb.pasteur.fr/cgi-bin/bigsdb/bigsdb.pl?db=pubmlst_bordetella_isolates&page=query&project_list=29&submit=1

iTOL interactive trees: https://itol.embl.de/shared/1l7Fw0AvKOoCF

Human research participants

Policy information about studies involving human research participants and Sex and Gender in Research.

Reporting on sex and gender	n/a
Population characteristics	n/a
Recruitment	n/a
Ethics oversight	n/a

Note that full information on the approval of the study protocol must also be provided in the manuscript.

Field-specific reporting

Please select the one below that is the best fit for your research. If you are not sure, read the appropriate sections before making your selection.

☒ Life sciences ☐ Behavioural & social sciences ☐ Ecological, evolutionary & environmental sciences

For a reference copy of the document with all sections, see nature.com/documents/nr-reporting-summary-flat.pdf

Life sciences study design

All studies must disclose on these points even when the disclosure is negative.

Sample size	For genus phylogeny, we selected only some genomes representatives of each sublineage within genomic species (n=92).<br>For B. bronchiseptica genomic species, we took all genomes avalaible (n=211).<br>For B. pertussis, we selected a few hundreds genomes to cover the main ptxP branches and included all genomes who displayed macrolide-resistance features (n=124).
Data exclusions	From the 2582 public entries found in BIGSdb Bordetella database, we choose to exclude genomes with lower quality (e.g. high number of contigs). For the selection of curated genomes used in our analyses, we were carefull to exclude duplications of isolates. This is important in phylogenetic reconstruction to avoid introducing bias and a disrupted phylogenetic signal.
Replication	Genomes are usually not made several times for the same isolates, unless obvious reason (e.g. improving genome quality, genome contamination, etc...). No replication were made.
Randomization	In our study, randomization only occurs in phylogenetic tree reconstruction to assess tree robustness and branch support (bootstrap values).<br>No experimental groups were made.
Blinding	This is a descriptive study of public data. No comparison between experimental groups was done.

Reporting for specific materials, systems and methods

We require information from authors about some types of materials, experimental systems and methods used in many studies. Here, indicate whether each material, system or method listed is relevant to your study. If you are not sure if a list item applies to your research, read the appropriate section before selecting a response.

Materials & experimental systems

n/a	Involved in the study
☒	☐ Antibodies
☒	☐ Eukaryotic cell lines
☒	☐ Palaeontology and archaeology
☒	☐ Animals and other organisms
☒	☐ Clinical data
☒	☐ Dual use research of concern

Methods

n/a	Involved in the study
☒	☐ ChIP-seq
☒	☐ Flow cytometry
☒	☐ MRI-based neuroimaging
