# Supplementary material for: A comprehensive resource for Bordetella genomic epidemiology and biodiversity studies
Source: Nat Commun. 2022 Jul 1;13:3807. doi: 10.1038/s41467-022-31517-8 (PMC9249784; doi:10.1038/s41467-022-31517-8)
Supplement: Supplementary file 4 — Description of Additional Supplementary Files [file 41467_2022_31517_MOESM4_ESM.pdf]

Title: Supplementary Data 1:
Description: ANI values across the *Bordetella* genus.
This table presents pairwise ANI values for one representative of each known species and proposed new genomic species.

Title: Supplementary Data 2:
Description: Full list of isolates of novel genomic species. This table presents the complete list of isolates belonging to each proposed genomic species with their provenance and accession numbers.

Title: Supplementary Data 3:
Description: cgMLST genome coverage.
A summary of genome fractions covered by each cgMLST schemes for each species.

Title: Supplementary Data 4:
Description: *Bordetella* genomes list and accession numbers.
This table is listing all isolates included in our analysis, the corresponding metadata, their ID within BIGSdb and accession numbers in NCBI and their belonging to either BIGSdb projects 23, 24 or 25. Genomes used for ANI analysis are also shown.

Title: Supplementary Data 5:
Description: Correspondence between former alleles from Oxford's PubMLST BIGSdb database, and the current alleles in the merged database.
A list that tracks the correspondence of allele between former PubMLST BIGSdb Bordetella database and the new one hosted at Institut Pasteur, for some genes linked to virulence and their length.

Title: Supplementary Data 6:
Description: Main alleles of the virulence-related schemes observed in the BbGS lineages.
This table presents a summary of the most frequently observed alleles for each BbGS lineages and for Bpp_{ov}.
